# HexA-Enzyme Coated Polymer Nanoparticles for the Development of a Drug-Delivery System in the Treatment of Sandhoff Lysosomal Storage Disease

**DOI:** 10.3390/jfb13020037

**Published:** 2022-03-31

**Authors:** Eleonora Calzoni, Alessio Cesaretti, Nicolò Montegiove, Alessandro Di Michele, Roberto Maria Pellegrino, Carla Emiliani

**Affiliations:** 1 Department of Chemistry, Biology and Biotechnology, University of Perugia, Via del Giochetto, 06123 Perugia, Italy; eleonoracalzoni@gmail.com (E.C.); nicolo.montegiove@gmail.com (N.M.); roberto.pellegrino@unipg.it (R.M.P.); carla.emiliani@unipg.it (C.E.); 2 Centro di Eccellenza sui Materiali Innovativi Nanostrutturati (CEMIN), University of Perugia, Via Elce di Sotto 8, 06123 Perugia, Italy; 3 Department of Physics and Geology, University of Perugia, Via Pascoli, 06123 Perugia, Italy; alessandro.dimichele@unipg.it

**Keywords:** lysosomal storage disorders, covalent immobilization, biopolymer nanoparticles, enzyme replacement therapy, restored activity, ganglioside degradation, Sandhoff disease

## Abstract

Lysosomal storage disorders (LSDs) are a set of metabolic diseases caused by mutations in genes that are in charge of the production of lysosomal enzymes, resulting in the buildup of non-degraded substrates and the consequent systemic damage that mainly involves the Central Nervous System (CNS). One of the most widely used and studied treatments is Enzyme Replacement Therapy, which is based on the administration of the recombinant deficient enzyme. This strategy has often proved fallacious due to the enzyme instability in body fluids and its inability to reach adequate levels in the CNS. In this work, we developed a system based on nanotechnology that allows a stable enzyme to be obtained by its covalent immobilization on nanoparticles (NPs) of polylactic acid, subsequently administered to a cellular model of LSDs, i.e., Sandhoff disease, caused by the absence or deficiency of the β-d-*N*-acetyl-hexosaminidase A (HexA) enzyme. The HexA enzymes, loaded onto the polymeric NPs through an immobilization procedure that has already been investigated and validated, were found to be stable over time, maintain optimal kinetic parameters, be able to permeate the plasma membrane, hydrolyze HexA’s natural substrate, and restore enzyme activity close to the levels of healthy cells. These results thus lay the foundation for testing the HexA-NPs in animal models of the disease and thus obtaining an efficient drug-delivery system.

## 1. Introduction

Lysosomal storage disorders (LSDs) are a group of metabolic disorders caused by mutations of genes encoding enzymes of the lysosomal compartment, whose deficiency or absence causes the accumulation of non-degraded substrates. This accumulation determines a series of clinical manifestations, many of which involve severe and progressive impairment of the Central Nervous System (CNS) functions, and as of today, there is no cure other than palliative and supportive therapies in most cases. Almost all LSDs occur in childhood, with very few exceptions that can manifest at an older age. Some of these pathologies have been treated with Enzyme Replacement Therapy (ERT), others with Gene Therapy (GT), but a definitive therapy has not been found yet, because of the impossibility for recombinant enzymes or viral vectors to come across the Blood-Brain Barrier (BBB) [1,2,3,4,5,6,7,8,9,10]. The BBB has the task of maintaining cerebral homeostasis and protecting the CNS from the passage of potentially harmful substances, thus representing an almost insuperable barrier for the administration of pharmaceutical agents [11]. Therefore, some studies are currently underway intending to provide recombinant enzymes through the BBB [12,13,14,15].

Nanotechnologies represent a valid way to overcome this problem; in fact, to date, numerous nanovectors, such as liposomes, dendrimers, or polymeric nanoparticles (NPs) have been tested for the transport of molecules or drugs [11,16,17,18,19,20]. Many polymers are used for the controlled release of drugs or biological molecules. Polylactic acid (PLA), polyglycolic acid (PGA), and poly lactic-co-glycolic acid (PLGA) are the most used materials for NP synthesis and are extremely interesting by virtue of their biocompatibility and biodegradability [12,21,22,23,24,25,26,27]. In particular, PLA represents also an eco-friendly solution as it is a biopolymer that derives from the fermentation of plant starch (usually corn), with a low environmental impact associated with its production [28]. NPs have dimensions ranging from some to a few hundreds of nanometers; however, those having radii of about 100 nm are those with the best pharmacokinetic properties as they are able to cross the body’s anatomical barriers, such as BBB and plasma membranes, without being eliminated by the renal filtration system [12,29]. The entry of NPs into cells is mediated by endocytosis mechanisms, such as phagocytosis, macropinocytosis, and receptor-mediated endocytosis [30].

The use of nanovectors such as NPs could therefore represent a valid method for carrying deficient enzymes within the body, thus bypassing some of the main problems associated with ERT, such as the instability of the recombinant enzyme in in vivo systems [31]. In fact, recombinant enzymes have a very short half-life in serum due to a rapid decrease in enzymatic kinetics, which means that there is little accumulation in pathological sites such as the CNS, so that they need to be administered frequently to maintain therapeutic levels [32,33,34]. Loading the NPs with a specific enzyme can indeed help to implement the ERT as by doing so, it is possible to evade the body’s defenses, overcome its anatomical barriers and increase the stability of the enzyme [34]. These nanosystems ensure greater physico-chemical and biological stability of the transported molecules and in this way, the intravenously injected enzymes reach the body’s organs more efficiently [14,15]. Enzymes’ stability is a major issue when dealing with their delivery and different immobilization techniques offer different advantages, for example, entrapment allows enzyme activity protection, while covalent immobilization, being characterized by strong bonding with the polymer surface, prevents enzyme molecules from leaching and endows them with high thermal stability [35,36]. As part of the enormous potential represented by drug delivery mediated by NPs, we developed a system consisting of polymeric NPs of polylactic acid (PLA) covered with a lysosomal enzyme, β-d-*N*-acetyl-hexosaminidase A, to evaluate the effectiveness of internalization and functioning of HexA-NP complex in cellular models of Sandhoff disease, one of the most serious disorders among all LSDs [37]. PLA was chosen because it is a biocompatible and biodegradable material, but especially by virtue of its long half-life when in contact with biological media. In fact, biocompatibility, biodegradability, and long half-life are the three key features that make PLA perfectly fit for biomedical applications [35]. Biocompatibility is necessary for a safe administration of the enzyme, biodegradability allows the polymer not to be accumulated in the body after it has carried out its task, and a long half-life is fundamental for the prolonged functionality of the enzyme-NP system. There are two main isoforms of the Hex enzyme in humans, the HexA isoform, a heterodimer consisting of α and β subunits, and the HexB isoform, a ββ homodimer. Between the two isoforms, HexA is the one that plays a more relevant physiological role, as it is the only one able to hydrolyze the GM2 ganglioside [38,39,40]. Mutations in the genes that code for the α or β subunit determine the absence of the HexA isoform and therefore an accumulation of this substrate inside the cells, with the subsequent impairment of the CNS, which is particularly rich in this ganglioside. In Sandhoff disease, a mutation in the HEXB gene determines the absence or abnormal functioning of the HexA enzyme [37]. This LSD is characterized by a progression of symptoms defined by systemic and neurological deficit which unfortunately leads to death within a few years of life [1,39,41]. There is currently no cure for this pathology, and although ERT has been the most used strategy, it has so far failed due to the instability of the recombinant enzyme administered and its inability to reach the CNS [42,43,44]. However, as already fully explained in our previous article [35], it is possible to develop a system in which the HexA enzyme is covalently immobilized on a solid support to increase its stability. In fact, the immobilization of the enzyme prevents its inactivation, potentially caused by multiple factors, and increases its half-life up to over a year. For this reason, our work aims at creating an enzymatic drug-delivery system based on the use of polymeric NPs made of PLA, which have been coated with the HexA enzyme in order to test them on in vitro models of the Sandhoff disease, by evaluating the restored activity of the enzyme within the cells and its ability to degrade the GM2 ganglioside. These findings lay the foundations for investigating an improved ERT for the treatment of LSDs.

## 2. Materials and Methods

### 2.1. Materials

Polylactic acid (PLA), Diethylenetriamine, 25% Glutaraldehyde stock solution, and trypan blue powder were purchased from Sigma-Aldrich (Saint Louis, MO, USA). Quick Start™ Bradford 1× Dye Reagent was purchased from Bio-Rad (Hercules, CA, USA). 4-Methylumbelliferyl-6-sulfo-2-acetamido-2-deoxy-β-d-glucopyranoside (MUGS) was purchased by Toronto Research Chemicals (Toronto, ON, Canada). Dulbecco’s modified Eagle’s medium (DMEM), fetal bovine serum (FBS), Trypsin, and Penicillin/Streptomycin were purchased from Euroclone (Pero, Italy). HexA polyclonal antibody and secondary antibody conjugated with Alexa Fluor^®^ 488 were purchased from Thermo Fisher Scientific (Waltham, MA, USA). Vectashield^®^ Vibrance™ Antifade Mounting Medium with 4′,6-diamidino-2-phenylindole (DAPI) was purchased from Vector Laboratories Inc. (Burlingame, CA, USA).

### 2.2. Synthesis of PLA Nanoparticles (NPs) 

The PLA powder was dissolved in chloroform at a concentration of 5% *w*/*v*. The solution was immersed in cold water at 4 °C and irradiated with high-power ultrasounds by an Ultrasonic processor VCX750 (Sonics & Materials, Inc., Newtown, CT, USA), operating at 20 kHz and at 40% of amplitude, 3 times for 15 s, with pauses of 10 s each. The solution containing the NPs was kept under magnetic stirring for 1 h at room temperature. After this period, the solution was centrifuged at 14,000× *g* for 20 min; finally, the NP pellet was recovered and abundantly washed with deionized water.

### 2.3. Immobilization of β-d-N-Acetyl-Hexosaminidase A 

The immobilization protocol is the object of an Italian Patent n. 102020000003344 and has previously been described elsewhere [35,45]. It involves a first functionalization step in order to cover the material with NH_2_ groups and a subsequent activation phase of the NPs with glutaraldehyde. For the NP functionalization, a diethylenetriamine solution was used after dilution in propanol (21% *v*/*v*) for 1 h at 55 °C. Afterward, NPs were activated with a 2.5% glutaraldehyde solution in deionized water, left to act for 3 h at room temperature. After removal of the glutaraldehyde solution and abundant washing of the PLA NPs with deionized water, the previously purified HexA enzyme [35] was immobilized, leaving 1 mL of the 50 µg/mL enzymatic solution in an overnight incubation at 4 °C. Each step of the functionalization/immobilization process was followed by centrifugation at 14,000× *g* for 20 min to form an NP pellet. The quantity of enzyme bound to the NPs was estimated through Bradford’s assay [46] by evaluating the difference between the initial protein concentration in the enzyme stock solution and the solution recovered after immobilization.

### 2.4. SEM and DLS Analysis

The morphology of the NPs was examined by Field Emission Gun Electron Scanning Microscopy (FE-SEM) LEO 1525 ZEISS (Carl Zeiss SMT AG, Oberkochen, Germany) after metallization with Cr. The acceleration potential voltage was maintained at 5 keV and measurements were carried out using an in-lens detector. 10 µL of an NP-containing solution were deposited on a silica layer and subsequently metalized. The analysis was carried out on both raw NPs and functionalized and activated NPs loaded with HexA. Dynamic Light Scattering (DLS) analysis was performed on freshly prepared HexA-NPs to evaluate the dimensions of NPs using a Nicomp 380 ZLS autocorrelator equipped with a He Ne Laser source at 632.8 nm (Particle Sizing System, Inc., Santa Barbara, CA, USA).

### 2.5. β-d-N-Acetyl-Hexosaminidase A Enzyme Assay

The activity of the HexA-NPs was measured using the specific synthetic fluorogenic substrate 4-methylumbelliferyl-β-*N*-acetylglucosaminide-6-sulfate (MUGS), as described in refs. [35,47]. The milliunits (mU) of the enzyme, where 1 mU is the amount of enzyme that hydrolyses 1 nmol of substrate/min at 37 °C, were calculated by referring to a calibration curve set up using 4-methylumbelliferone at different concentrations. Enzymatic activity for the HexA-NPs was calculated as mU of enzyme per g of PLA. Preliminary tests showed there is a linear relation between product release and time, at least up to 30 min, under all the experimental conditions employed, allowing fluorescence intensity to be quantitatively correlated with the mU of enzymatic activity in all cases. 

### 2.6. Biochemical Characterization of HexA-NPs

Stability towards pH was tested using the MUGS substrate in a pH range between 2 and 9 [35]. The Km value of HexA-NPs was determined using the linear transformation of Lineweaver and Burk. The samples were incubated with the artificial MUGS substrate in a 0.06–2.0 mM concentration range for 15 min at 37 °C. Km value is expressed as concentration units (mM). The thermal stability of HexA-NPs was tested as a function of time by bringing the enzyme to the temperature of interest and waiting for increasing times. The activity towards MUGS was later studied at 37 °C, revealing whether different temperatures had caused any loss of activity. Finally, the temperature optimum of immobilized HexA was evaluated by using the MUGS substrate in a temperature range going from 6 °C to 70 °C, as previously detailed in ref. [35]. 

### 2.7. Sandhoff Cell Culture and HexA-NP Treatment

Human fibroblasts from an anonymous Sandhoff patient, taken before 2002, were offered for previous studies by Cell Line and DNA Biobank from patients affected by Genetic Disease of Istituto Giannina Gaslini of Genova (Italy) [48]. These particular Sandhoff cells feature a genotype characterized by a missense mutation in the gene coding for the β subunit of the Hex enzymes, which leads to the formation of an unstable mRNA [49,50]. The cells were cultured in DMEM medium containing 10% (*v*/*v*) heat-inactivated FBS and Penicillin 10,000 U per mL/Streptomycin 10 mg per mL. For the treatment with HexA-NPs, 1000 µL of DMEM culture medium containing 5 × 10^4^ Sandhoff cells were plated in a Falcon^®^ 12-well clear flat-bottom multiwell cell culture plate (Becton, Dickinson and Company, Franklin Lakes, NJ, USA) and incubated in 5% CO_2_ at 37 °C. The cell viability was monitored by trypan blue dye staining, using an automated cell counter (Invitrogen™ Countess™, Thermo Fisher Scientific, Waltham, MA, USA). After 24 h, the medium was replaced with 1000 µL of new medium containing 5 µg of the HexA enzyme conjugated to the NPs in a ratio of 150 µg/g of HexA relative to the PLA weight. After treatment, the cells were washed with PBS and lysed with 10 mM Na/P buffer pH 6.0 with 0.05% Triton X-100. The presence of the enzyme in the cell lysate was verified by testing the activity toward MUGS as described in paragraph 2.5. Specific activity (mU/mg) was expressed as mU of enzyme per mg of total proteins.

### 2.8. Immunofluorescence Assay

About 1 × 10^3^ Sandhoof cells and human fibroblast (HF) cells as an internal control were seeded on round glass coverslips previously sterilized by 30 s of immersion in 70% ethanol, rinsed with sterile phosphate buffer saline (PBS), and placed in a Falcon^®^ 24-well clear flat-bottom multiwell cell culture plates (Becton, Dickinson and Company, Franklin Lakes, NJ, USA). The cells were then incubated for 45 min in a humidified atmosphere with 5% CO_2_ at 37 °C; at the end of the incubation time, 500 µL of DMEM medium were gently added to each well and incubated for 24 h under canonical culture conditions (humidified atmosphere with 5% CO_2_ at 37 °C). Then, 500 µL of a HexA-NP solution corresponding to an amount of 0.25 µg of the HexA enzyme conjugated to the NPs diluted in DMEM were administered to Sandhoff cells and incubated for 24 h in a humidified atmosphere with 5% CO_2_ at 37 °C. Cells on round glass coverslips were then rinsed twice with PBS and fixed in 4% paraformaldehyde for 20 min at room temperature, and, after PBS rinsing, permeabilized (PBS + 3% FBS + 0.5% Triton X-100) and blocked (PBS + 3% FBS + 0.05% Triton X-100) for 1 h at room temperature. In order to carry out immunocytochemistry experiments, samples were incubated overnight at 4 °C with primary human antibody anti-HexA, followed by incubation with secondary antibody conjugated with Alexa Fluor^®^ 488 for 1 h at room temperature. After washing with PBS, samples were mounted, and nuclei were stained with Vectashield^®^ Vibrance™ Antifade Mounting Medium with 4′,6-diamidino-2-phenylindole (DAPI) [51]. Image acquisitions were performed by using a fluorescence microscope (Eclipse-TE2000-S, Nikon, Tokyo, Japan) equipped with the F-ViewII FireWire camera (Olympus Soft Imaging Solutions GmbH, Münster, Germany) and through the use of Cell^F^ Imaging Software (Olympus Soft Imaging Solutions GmbH, Münster, Germany). 

### 2.9. GM3 Analysis by Quadrupole Time-of-Flight Liquid Chromatography/Mass Spectrometry (Q-TOF LC/MS)

In order to evaluate the ganglioside GM2 degradation, the natural substrate of HexA, GM3 ganglioside semiquantification was performed by Liquid Chromatography coupled with High-Resolution Mass Spectrometry (LC/HRMS) using an Agilent 6530 Accurate-Mass Q-TOF LC/MS system (Agilent Technologies, Inc., Santa Clara, CA, USA) to detect GM3 ganglioside molecular species in the positive mode. Analyses were carried out using the method described by Koelmel J.P. et al. (2020) [52], adapted to the different instrumental configurations. The preparation of the sample was carried out with the MMC method [53]. Each sample was prepared in triplicate and run three times. Ganglioside annotation was performed by comparing accurate mass measurements with both LIPID MAPS (http://www.lipidmaps.org/tools/ms, accessed on 14 May 2021) and Human Metabolome Database (http://www.hmdb.ca/spectra/ms/search, accessed on 14 May 2021) prediction tools and confirmation came from the UltrafleXtreme LIFT-TOF/TOF mode on-tissue tandem measurements. MassHunter Acquisition B.09.01 and MassHunter Qualitative Analysis B.09.01 monitored the measurements and post-run analyses.

### 2.10. Statistical Analysis

Data shown in this study are reported as mean values of three samples studied of each cellular model tested (i.e., healthy HFs as CTRL+, untreated Sandhoff cells as CTRL−, and HexA-NP treated Sandhoff cells) ± standard error of the mean (SEM). The Student’s *t*-test was used to analyze the significance of the specific activity differences between the means of the CTRL+ and CTRL− against the HexA-NP treated Sandhoff cells. The Student’s *t*-test was also used to evaluate the significance of the differences in the GM3 ganglioside species content assessed by Q-TOF LC/MS between the means of the CTRL+ and CTRL− against the HexA-NP treated Sandhoff cells. The level of significance for the data was set at *p* < 0.05. All statistical tests were done using GraphPad Prism 9.00 for Windows (GraphPad Software, San Diego, CA, USA).

## 3. Results and Discussion

### 3.1. HexA Immobilization on PLA NPs and Morphological Analysis

**Figure 1:**
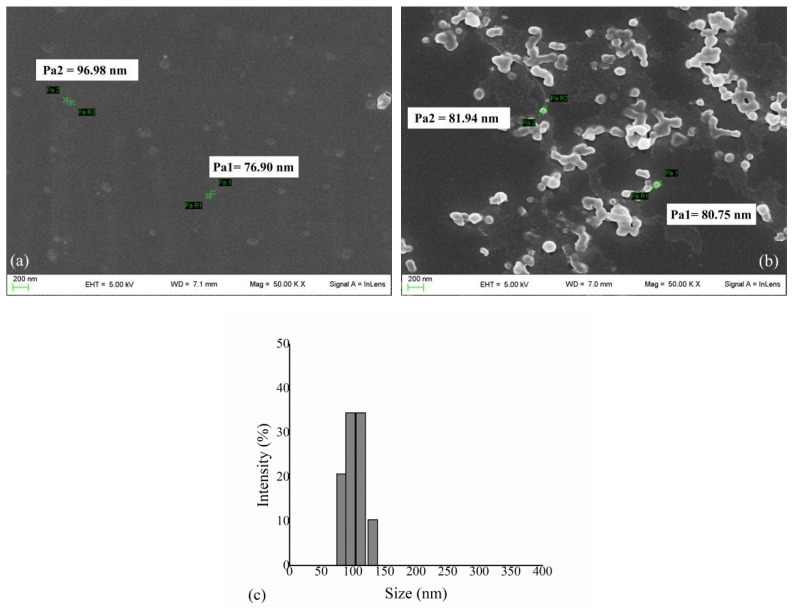
(**a**) SEM analysis of PLA NPs and (**b**) HexA-conjugated PLA NPs. The images were obtained with a magnification of 50,000× and show the characteristic sizes of some NPs. (**c**) DLS analysis of HexA-PLA NPs showing size distribution.

The recombinant enzyme HexA was produced and purified according to the methods described in a previous work [35] and subsequently immobilized on PLA NPs by the patented procedure presented therein. The immobilization of the enzyme on PLA NPs was verified by the Bradford assay and an immobilization percentage of 90% was recorded. The enzyme concentration relative to the weight of the final biomaterial ranged from 100 µg/g to 250 µg/g. The morphology of PLA NPs and PLA NPs coated with the HexA enzyme was analyzed by Scanning Electron Microscopy (SEM) (Figure 1).

The images obtained by SEM have revealed that the NPs size ranges between 40 and 150 nm, with an average diameter of 85 nm (Figure 1a); after the functionalization/activation and immobilization processes have occurred (Figure 1b), the same dimensions can be retrieved for isolated NPs, which are detectable together with larger aggregates (Figure 1b). The dimensions of HexA-NPs were also investigated through DLS analysis, which corroborated the results of SEM, showing that the diameter of NPs ranges from about 80 to 130 nm, with a mean value of 102 nm (Figure 1c). NPs with dimensions up to 70–150 nm represent excellent carriers for the delivery of therapeutic molecules, in that, by virtue of their size, they are able to evade the body’s defenses and eventually cross anatomical barriers including the plasma membrane and the BBB [12,54,55,56,57].

### 3.2. HexA-NP Stability

**Figure 2:**
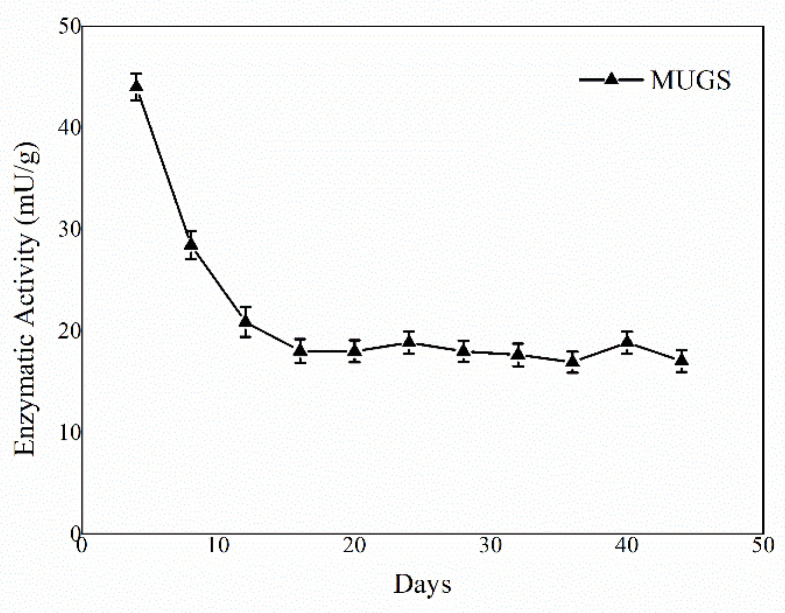
Trend of the enzyme activity of HexA immobilized on PLA NPs against the artificial substrate MUGS as a function of time. Data are reported as mean ± SEM, *n* = 3.

The activity of the immobilized HexA molecules was tested against the artificial substrate MUGS, which is specifically hydrolyzed only by this isoform of the enzyme, and the assay was repeated periodically under the same conditions in order to assess the stability over time of the enzyme-PLA NP system. The enzymatic assay was repeated on three different PLA NP preparations and the results are reported as mean values. The revelations were performed for more than forty days after the immobilization event and the mean results concerning the NP preparations are shown in Figure 2.

The first cycles showed high enzymatic activity, about 45 mU/g, subsequently, a decrease in enzymatic activity was detected supposedly due to a partial deactivation of the enzyme in agreement with the behavior detected for HexA immobilized on PLA films, as described in a previous paper [35]. Following the third revelation, an apparent stabilization of the enzymatic activity has been highlighted, confirming that the HexA enzyme immobilized on PLA NPs is a long-term stable system that maintains about 40–45% residual activity (almost 20 mU/g) relatively to the initial value, for several days during numerous passages, without undergoing further reduction.

### 3.3. Biochemical Characterization of HexA-NPs

**Figure 3:**
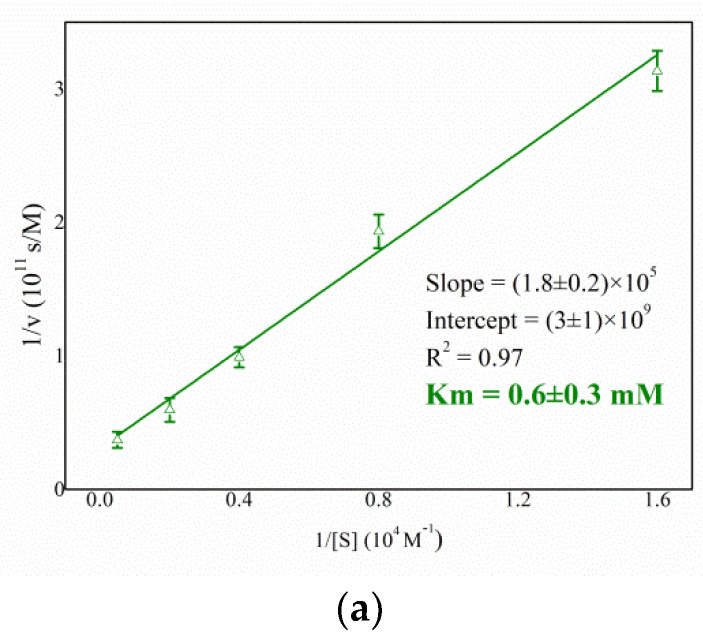

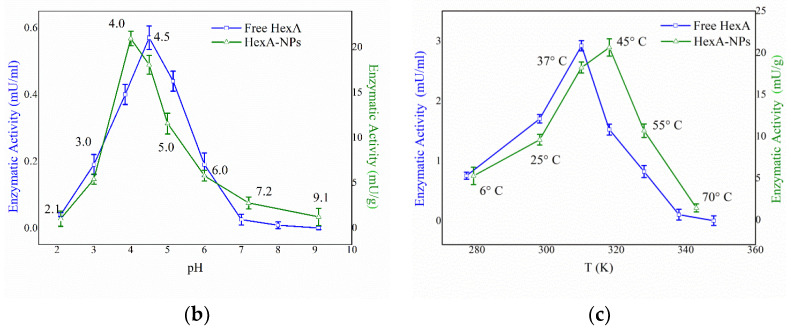
Km analysis (**a**), pH effect (**b**), and temperature effect (**c**) on the enzymatic activity of HexA-NPs toward the artificial substrate MUGS. Data are reported as mean ± SEM, *n* = 3.

An accurate biochemical analysis was conducted to compare the function and stability of the HexA enzyme in its immobilized forms on PLA NPs relatively to its free form [35]. The Km value, pH, and temperature effects were tested at different times after immobilization and the mean values of the different measurements carried out are reported in Figure 3.

Figure 3 shows that the immobilization procedure does not modify the biochemical characteristics of the enzyme in its immobilized form. The Km value (Figure 3a) of the immobilized enzyme (0.6 ± 0.3 mM) remains similar to that of free form (1.0 ± 0.5 mM) [35] indicating that the immobilization process does not alter the affinity towards the substrate. Additionally, the HexA-NPs maintain an optimum at acidic pH as its free form, around a value of 4.0, characteristic of lysosomal hydrolases (Figure 3b). Furthermore, in a previous study, the thermal stability of HexA was evaluated revealing that while the enzyme in its free form is a highly thermolabile molecule, in its immobilized form it is particularly thermostable up to 4 h at 70 °C [35]. In fact, the temperature optimum study revealed that the HexA-NPs were significantly active even at temperatures above the physiological one (Figure 3c), with the optimum shifting from 37 °C to 45 °C. These results suggest that HexA-NPs are a stable system capable of preserving all the biochemical characteristics of the enzyme in its free form. The bond with the NPs indeed stabilizes the HexA activity over time, as well as their resistance to temperature, characteristics which are very important for any drug-delivery application.

### 3.4. Immunofluorescence Assay 

The ability of the HexA-NP system to enter the cell and to deliver an acceptable amount of enzyme was evaluated by immunofluorescence analysis on a pathological cell line of Sandhoff disease, using untreated pathological Sandhoff cells as negative controls (CTRL−), and healthy human fibroblast (HF) cells as internal positive controls (CTRL+). 

**Figure 4:**
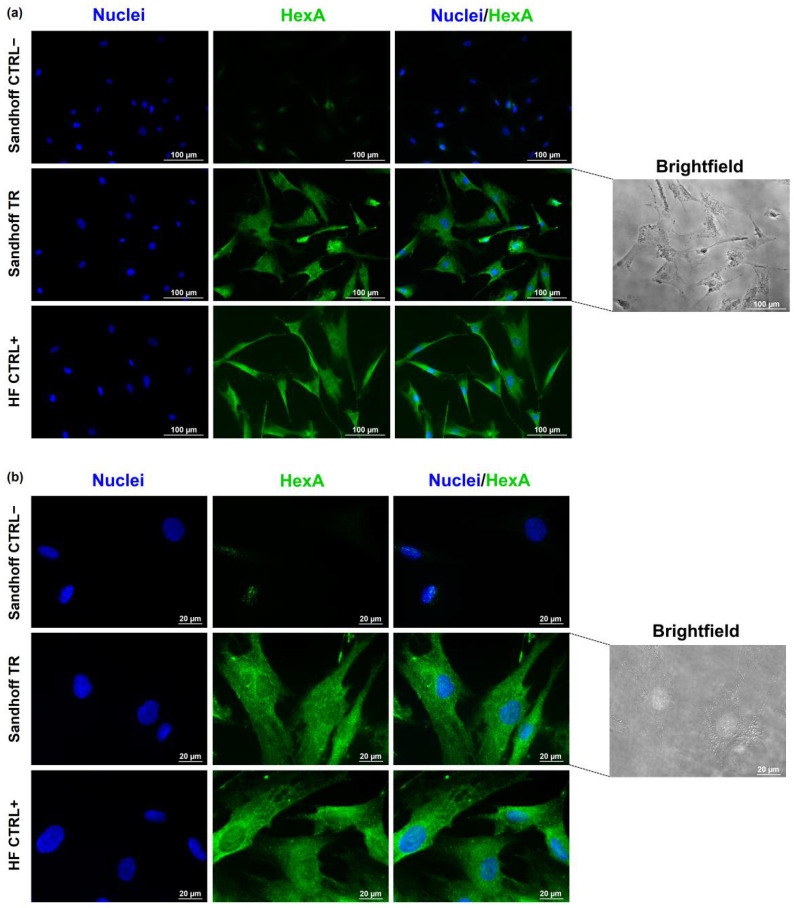
Representative fluorescence images of nuclei (DAPI, blue) and HexA (Alexa Fluor^®^ 488, green) in untreated Sandhoff cells (CTRL−), HexA-NP treated Sandhoff cells (TR), and healthy human fibroblast (HF) cells (CTRL+) at an objective magnification of 20× (panel (**a**)), and of 60× (panel (**b**)). On the right side of the panels, brightfield images of HexA-NP treated Sandhoff cells (TR) are shown at magnification 20× (panel (**a**)) and 60× (panel (**b**)).

Figure 4 shows that the HexA enzyme is almost absent in the untreated Sandhoff cells (CTRL−), the recognition of some scattered dots after the anti-HexA antibody exposure is likely due to the mutation of this cell line, which determines the production of unstable mRNAs and thus the presence of only slight traces of the protein. The image shows instead how the treatment with the HexA-NP system is effective: the treated Sandhoff cells (TR) exhibit a correlated-HexA fluorescence intensity almost identical to that of the cells used as a positive control (HFs, CTRL+). This data is also supported by brightfield images in which the presence of the NPs inside the cells is clearly evident proving the efficiency of this carrier system. 

### 3.5. In Vitro Tests with HexA-NPs

**Figure 5:**
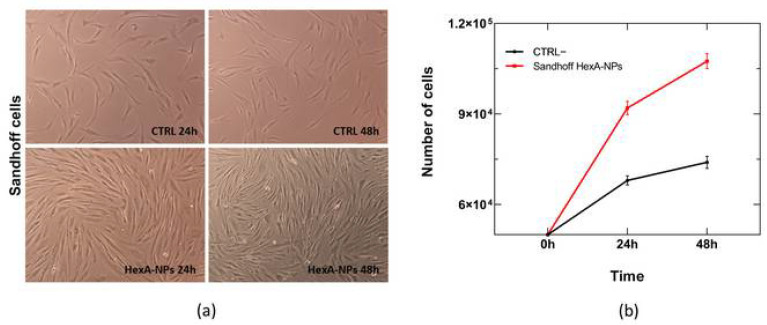
Images in brightfield (10× objective magnification) of untreated Sandhoff cells (CTRL−) and HexA-NP treated Sandhoff cells at 24 and 48 h (panel (**a**)) and relative growth curves (panel (**b**)). Data are reported as mean ± SEM, *n* = 3.

In order to evaluate the functioning of the enzymes linked to the PLA NPs, HexA-NPs were tested in vitro on the same pathological cell line of Sandhoff disease. HexA-NPs were administered to the cells ten days after their preparation so that the activity of the immobilized enzyme was not expected to reduce with time, as already described in Section 3.2, Figure 2. The effect of HexA-conjugated NPs on the cells was assessed by optical microscopy after 24 and 48 h. Cell viability was evaluated by cell count with trypan blue dye so that it was possible to plot a growth curve for the treated Sandhoff cells and the untreated culture, taken as the negative control. Figure 5 shows that the treatment does not damage the cell line, whose morphology remains unchanged compared to control cells after 24 and 48 h. Furthermore, treatment with enzyme-loaded NPs also stimulates cell growth compared to pathological controls, indicating how the administration of the deficient enzyme benefits the overall metabolism of diseased cells.

**Figure 6:**
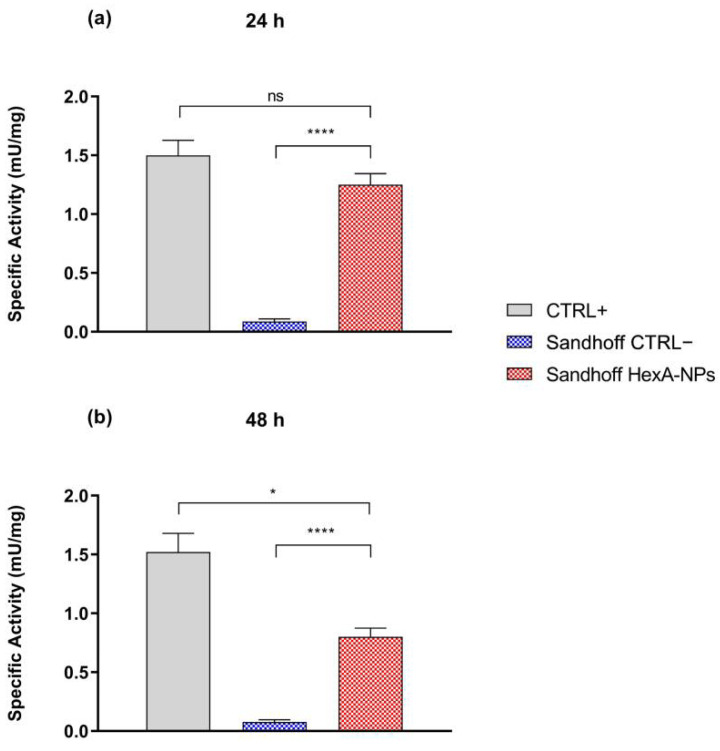
Specific activity (mU/mg) toward the artificial substrate MUGS of healthy human fibroblasts (CTRL+), untreated Sandhoff cells (CTRL−), and HexA-NP treated Sandhoff cells after 24 h (panel (**a**)) and 48 h of treatment (panel (**b**)). Data are reported as mean ± SEM, *n* = 3. ns = difference is not statistically significant, * *p* < 0.05, **** *p* < 0.0001.

The NP uptake was also evaluated by enzymatic assay with the artificial substrate MUGS on the cell lysates to verify the effective presence and activity of the enzyme administered to the cells thanks to the binding to the NP carrier. The specific activity values toward the substrate after 24 and 48 h of NP treatment in Sandhoff cells, compared to healthy HF cells (CTRL+) and untreated Sandhoff cells (CTRL−), are shown in Figure 6 and Table 1.

In order to verify the efficient HexA-NP uptake by the cells, the medium was also investigated in the search for the enzyme, but no residual activity was detected independently of the incubation time.

In the untreated Sandhoff cells (CTRL−), where the gene for the β subunit is mutated, the isoform HexA (αβ) is severely deficient, and the activity towards the substrate is nearly zero. On the other hand, HexA-NP treated Sandhoff cells show a significant increase in specific activity against the substrate, very close to the activity shown by healthy cells (CTRL+). In particular, the activity against MUGS increases compared to the untreated Sandhoff cells by a factor approaching fifteen after 24 h of treatment, bringing the activity of the HexA enzyme back to about 83% of that shown by healthy HFs (CTRL+). After 48 h the activity of the treated Sandhoff cells is still increased by over one order of magnitude compared to the untreated Sandhoff cells, but with an enzyme activity value of about 55% compared to the HFs. This behavior is attributable to cell division that dilutes the initial concentration of the administered HexA-NPs (Figure 6 and Table 1): as cells duplicate, although the overall amount of functional enzyme inside treated cells remains unchanged, the cellular protein content duplicates as well, and the specific activity (calculated as mU of enzyme per mg of total proteins) is condemned to reduce.

The rate of decay of the specific enzymatic activity with the growth of the cell population was also evaluated for several days and cell division cycles in order to understand how long it is possible to maintain an acceptable activity value of functional enzyme inside the cells. 

**Figure 7:**
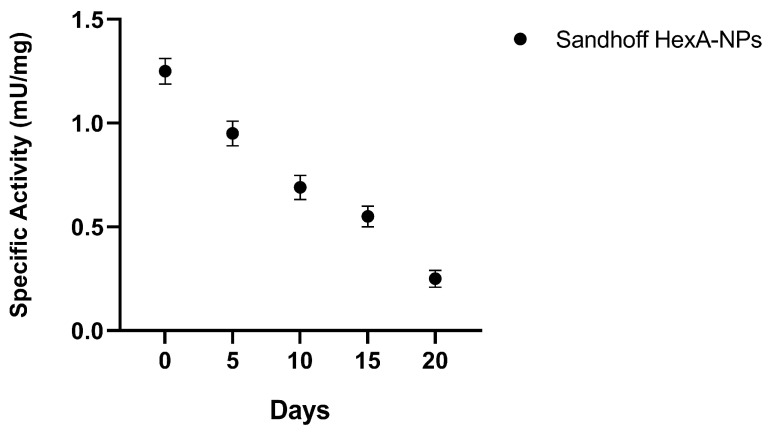
Specific activity (mU/mg) over time toward the artificial substrate MUGS of HexA-NP treated Sandhoff cells. Data are reported as mean ± SEM, *n* = 3.

As shown in Figure 7, after the initial administration, the activity decreases almost linearly with time with decay to almost half of the initial value after 10 days. This data is useful for understanding how often a new administration of the deficient enzyme is required under the conditions employed to have an adequate amount of HexA inside the cells. In particular, a second administration of HexA-NPs after 20 days would restore the enzymatic activity in the investigated in vitro culture to a value close to its normal level of 1.5 mU/mg. However, it has to be stressed that this only applies to in vitro fibroblasts, as the physiology of cells in vivo would be totally different. In fact, cells in vitro are constantly added with large quantities of growth factors that do not correspond to those normally found under physiological conditions. Conversely, cells in vivo undergo slower growth, especially the cells of the nervous system which are mostly considered incapable of entering cell cycle and proliferating. In vivo the dilution of the administered enzyme would therefore be slowed down as well, allowing the frequency of successive enzyme-NP administrations to be definitely more sporadic. 

### 3.6. GM3 Analysis by Quadrupole Time-of-Flight Liquid Chromatography/Mass Spectrometry (Q-TOF LC/MS)

**Figure 8:**
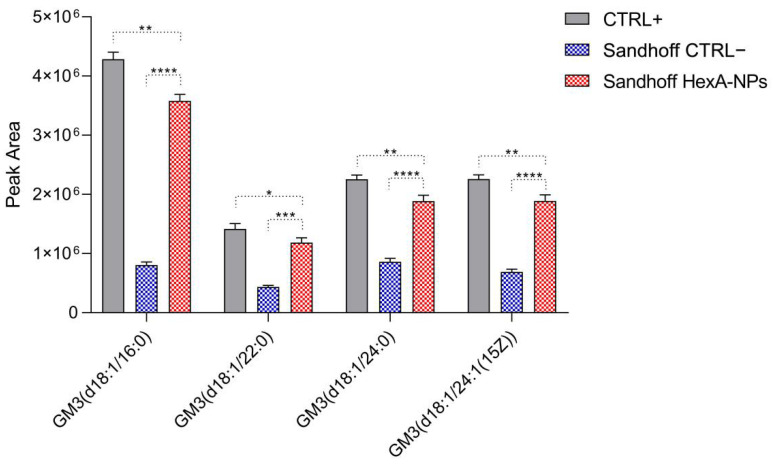
Main GM3 species detected by Q-TOF LC/MS analysis in healthy human fibroblasts (CTRL+), untreated Sandhoff cells (CTRL−), and HexA-NP treated Sandhoff cells. Data are reported as mean ± SEM, *n* = 3. * *p* < 0.05, ** *p* < 0.01, *** *p* < 0.001, **** *p* < 0.0001.

The effective in vitro functioning of the HexA-NP system was then evaluated in terms of the ability of the administered enzyme to act against its natural substrate: the GM2 ganglioside. Q-TOF LC/MS analysis was thus carried out to search for the presence of different GM3 ganglioside species produced upon GM2 degradation in the treated cells after 24 h. Figure 8 shows the abundance histograms of the main GM3 ganglioside species detected in healthy HFs (CTRL+), untreated Sandhoff cells (CTRL−), and HexA-NP treated Sandhoff cells.

Figure 8 shows that after treatment with the HexA-NP system there is a significant increase in the abundance of four major GM3 ganglioside species compared to the untreated Sandhoff cells, with the GM3 ganglioside (d18:1/16:0) being certainly the most abundant among these species, in the HexA-NP treated Sandhoff cells. As with the artificial substrate MUGS, also for the natural ganglioside substrate, the abundance of the different GM3 species in the treated Sandhoff cells is comparable to HF cells (CTRL+). This result confirms those previously shown (Figure 5 and Table 1) and further demonstrates that the HexA-NP system is able to enter the cells and favor a restoration of the enzymatic activity, determining the hydrolysis of the accumulated GM2 ganglioside to the GM3 species.

## 4. Conclusions

This work describes a system consisting of the recombinant HexA enzyme immobilized on polylactic acid (PLA) nanoparticles (NPs), using a procedure that we have previously developed and patented, for the development of a drug-delivery system in the treatment of Sandhoff Lysosomal Storage Disease. The immobilization process led to a stable enzyme, capable of retaining all the biochemical characteristics of the free enzyme. Moreover, this strategy provides an enzyme anchored on the surface of the NPs, readily available to carry out its function without needing to be released, as it instead happens when dealing with encapsulated enzymes. An effective internalization of the HexA-NP system within Sandhoff cells emerged from in vitro experiments. Immunocytochemistry proved how the treatment determines the cellular uptake of the deficient enzyme, whose levels within the diseased cells return comparable to those of the healthy control cells. An actual internalization and a significant restoration of the HexA enzyme activity in the treated Sandhoff cells were also corroborated both by enzymatic assays with an artificial substrate and by evaluation of the formation of the GM3 ganglioside, produced by the hydrolysis of the natural substrate GM2, as revealed by Q-TOF LC/MS analysis. Further to this, the cellular uptake of the HexA-NPs did not cause any toxic effect on the treated cells, whose vitality indeed benefited from the treatment. The ability of the HexA-coated-NPs to pass across plasma membranes and that of the exogenous enzyme to hydrolyze the GM2 ganglioside once in the cells provide concrete proof of the great potential of this system. These findings lay a sound foundation for carrying out future in vivo tests on animal models of Sandhoff disease, in order to verify the distribution of HexA-NPs in the body, including the CNS, and to understand its efficacy as a valuable drug-delivery system.

## 5. Patents

The above-described immobilization protocol is patented (Italian Patent n. 102020000003344 of 8 March 2022).

## Figures and Tables

**Table 1:** Specific activity (mU/mg) toward the artificial substrate MUGS of healthy human fibroblasts (CTRL+), untreated Sandhoff cells (CTRL−), and HexA-NP treated Sandhoff cells after 24 and 48 h. Data are reported as mean ± SEM, *n* = 3.

Specific Activity (mU/mg)	CTRL+	CTRL−	Sandhoff HexA-NPs
24 h	1.5 ± 0.1	0.09 ± 0.02	1.25 ± 0.09
48 h	1.5 ± 0.2	0.08 ± 0.02	0.85 ± 0.07

## Data Availability

The data presented in this study are available on request from the corresponding author.
